# Beyond the Surface: Mechanistic Intersections Between Obesity and UVB Exposure

**DOI:** 10.1038/s41366-026-02084-5

**Published:** 2026-04-08

**Authors:** Umar A. Sheikh, Tasduq A. Sheikh

**Affiliations:** 1 McArdle Laboratory for Cancer Research, Department of Oncology, The School of Medicine and Public Health, University of Wisconsin-Madison, Madison, WI USA; 2 Pharmacology Division, Council of Scientific and Industrial Research-Indian Institute of Integrative Medicine, Jammu, Jammu and Kashmir India; 3 Academy of Scientific and Innovative Research (AcSIR), Ghaziabad, India

**Keywords:** Cancer, Endocrinology, Risk factors

## Abstract

Obesity, a globally prevalent metabolic disorder, and ultraviolet-B (UVB) radiation, an inherent component of solar exposure, are each independently linked to a wide range of chronic health outcomes, including cancer, metabolic dysfunction, and immune dysregulation. Although traditionally viewed as separate risk factors, metabolic and environmental, respectively, emerging evidence reveals mechanistic intersections that may amplify disease burden when both are present. Obesity promotes chronic low-grade inflammation, oxidative stress, adipokine imbalance, and impaired DNA repair capacity, while UVB radiation induces mutagenic photoproducts, reactive oxygen species, and localized immunosuppression. These overlapping pathways converge on genomic instability, altered immune tolerance, and the development of tumor-promoting microenvironments, particularly within the skin. This review synthesizes recent advances in understanding the biological mechanisms linking obesity and UVB exposure, with emphasis on synergistic effects on oxidative stress, nucleotide excision repair efficiency, and immune surveillance. It examines experimental and clinical evidence supporting these interactions and highlights emerging therapeutic strategies, including phototherapy and vitamin D modulation, clarifying their mechanistic rationale in restoring immune balance and enhancing DNA repair. Finally, the review discusses implications for integrated prevention, risk stratification, and public health policy. By framing obesity and UVB radiation as a compound risk factor, this article underscores the need for interdisciplinary approaches to mitigate their combined impact on global health.

**Figure:**
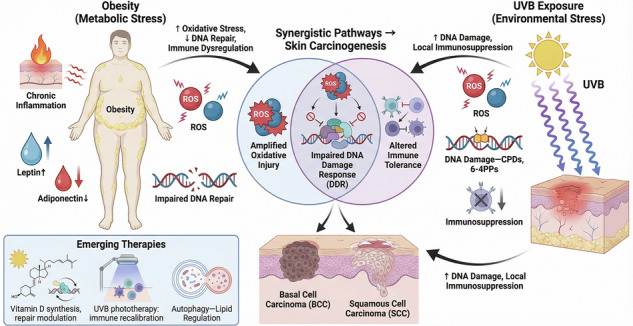
**Converging Effects of Obesity and UVB Exposure on Skin Carcinogenesis:** Obesity and UVB exposure converge through shared inflammatory, oxidative, and immune-modulating pathways that amplify skin cancer risk. Obesity drives chronic inflammation, oxidative stress, altered adipokine signaling, and impaired DNA repair, while UVB radiation causes direct DNA damage, reactive oxygen species, and local immunosuppression. Together, these stressors synergistically intensify oxidative injury, weaken DNA damage responses, and diminish immune surveillance, promoting the development of basal and squamous cell carcinoma. Emerging therapeutic approaches including vitamin D modulation, UVB-based phototherapy, and autophagy-targeted interventions, highlight opportunities for prevention and clinical translation.

**Converging Effects of Obesity and UVB Exposure on Skin Carcinogenesis:** Obesity and UVB exposure converge through shared inflammatory, oxidative, and immune-modulating pathways that amplify skin cancer risk. Obesity drives chronic inflammation, oxidative stress, altered adipokine signaling, and impaired DNA repair, while UVB radiation causes direct DNA damage, reactive oxygen species, and local immunosuppression. Together, these stressors synergistically intensify oxidative injury, weaken DNA damage responses, and diminish immune surveillance, promoting the development of basal and squamous cell carcinoma. Emerging therapeutic approaches including vitamin D modulation, UVB-based phototherapy, and autophagy-targeted interventions, highlight opportunities for prevention and clinical translation.

## Introduction

Obesity has emerged as one of the most significant global health challenges, with recent estimates revealing that hundreds of millions of adults worldwide are either people with obesity or living in a state of obesity [1], and prevalence continues to rise across all socioeconomic strata. Characterized by excess adiposity and systemic metabolic disruption, obesity is not simply an energy imbalance but a chronic, multifactorial condition defined by low-grade inflammation, oxidative stress, and immune dysregulation [2, 3]. These pathophysiological alterations extend well beyond metabolic disease, influencing cancer susceptibility, tissue repair, and immune surveillance [4–6]. A growing body of evidence highlights the impact of obesity-associated molecular mediators including the adipokines leptin and adiponectin; pro-inflammatory cytokines TNF-α and IL-6; and metabolic stress-driven alterations in nucleotide excision repair (NER) and base excision repair (BER) in shaping systemic and cutaneous immune responses [7, 8]. Leptin excess chronically activates JAK/STAT and NF-κB signaling, promoting persistent inflammation and skewing immune cell differentiation toward pro-tumorigenic phenotypes, whereas reduced adiponectin diminishes anti-inflammatory and antioxidant defenses [9]. Elevated TNF-α and IL-6 further disrupt immune homeostasis by altering dendritic cell maturation, suppressing regulatory T cell function, and enhancing reactive oxygen species (ROS) production, collectively weakening cutaneous barrier immunity [10, 11]. In parallel, obesity-induced oxidative stress and lipid peroxidation interfere with DNA repair regulators such as p53, XPC, and OGG1, reducing NER and BER efficiency and increasing vulnerability to UV-induced mutagenesis [12]. Together, these molecular disturbances create a systemic environment that fosters genomic instability, impaired immune surveillance, and heightened inflammatory signaling.

Ultraviolet-B (UVB) radiation (280–320 nm) is the most significant environmental risk factor for non-melanoma skin cancers (NMSCs), including basal cell carcinoma and squamous cell carcinoma [13–15]. UVB exposure induces direct DNA lesions such as cyclobutane pyrimidine dimers and 6-4 photoproducts, generates ROS, and promotes local immunosuppression, all of which contribute to carcinogenesis [16, 17]. Traditionally viewed as separate risk domains metabolic (obesity) versus environmental (UVB), emerging evidence suggests these factors may converge through shared pathways involving oxidative stress amplification, impaired DNA damage responses, and altered immune function. Obesity-driven inflammation and hormonal imbalances may compromise cutaneous defense mechanisms [18], whereas UVB-induced genotoxic stress can exacerbate systemic immune dysfunction, collectively heightening cancer susceptibility and potentially influencing metabolic outcomes [19, 20]. Dysregulation of ROS-mediated signaling, p53-dependent DNA repair, regulatory T cell function, and dendritic cell activity may represent key points of mechanistic overlap. Therapeutic insights further support this interplay. Phototherapy, especially narrowband UVB, can recalibrate dysregulated immune pathways by inducing tolerogenic dendritic cells, expanding regulatory T cells, and shifting cytokine profiles toward an anti-inflammatory state [21–23]. These effects may counteract obesity-associated immune activation and partially restore cutaneous immune homeostasis. Additionally, vitamin D, whose synthesis depends on UVB exposure, plays critical roles in epithelial barrier maintenance, antimicrobial peptide regulation, and p53- and NER-mediated DNA repair [24, 25]. Obesity-associated vitamin D deficiency may therefore worsen UVB-induced genotoxic stress and immune vulnerability, whereas targeted supplementation may help mitigate these effects. Together, these mechanistic insights emphasize how obesity and UVB exposure intersect to shape genomic stability and immune function. This review synthesizes current epidemiological, mechanistic, and experimental evidence on this interplay, highlights convergent pathways affecting skin cancer risk, and discusses emerging therapeutic strategies. We further outline the implications for integrated prevention, risk stratification, and public health policy as these two global health challenges continue to rise in parallel.

## Epidemiology and risk overlap

### Global burden of obesity

Obesity has evolved into one of the most pervasive and complex public health challenges of the 21st century. According to the World Obesity Atlas 2025, projections indicate that more than 1.1 billion adults will be living with obesity by 2030, reflecting a trajectory driven by rapid urbanization, dietary transitions toward energy-dense, nutrient-poor foods, and increasingly sedentary lifestyles [26]. Current WHO estimates reveal that nearly 890 million adults worldwide are already people with obesity [27], with prevalence accelerating across all income levels and geographic regions, underscoring its status as a global epidemic. The burden of obesity extends beyond individual health, exerting profound economic and societal impacts. It is a major risk factor for type 2 diabetes, cardiovascular disease, non-alcoholic fatty liver disease, and multiple cancers, including those of the breast, colon, and pancreas [28–30]. Importantly, obesity is now recognized as a systemic inflammatory condition, characterized by chronic low-grade inflammation, oxidative stress, and hormonal dysregulation, which collectively impair immune function and genomic stability [31–33]. In the United States, the situation is equally concerning. Data from NHANES (2021–2023) indicate that adult obesity prevalence has reached ~40%, with severe obesity approaching 10%, marking a steady upward trend over the past decade [34]. These figures highlight not only the scale of the problem but also its persistence despite ongoing public health interventions. Disparities remain stark, with higher prevalence observed among certain racial and ethnic groups, individuals with lower socioeconomic status, and populations residing in regions with limited access to healthy food and safe environments for physical activity [35–37]. Taken together, these trends position obesity as a dominant metabolic exposure contributing to global morbidity and mortality. Its intersection with other risk factors, raises critical questions about compound risk profiles, synergistic mechanisms, and the need for integrated prevention strategies that address both metabolic and environmental determinants of health.

### UVB exposure and skin cancer burden

Ultraviolet B (UVB) radiation (280–320 nm) is the most significant environmental carcinogen driving the development of non-melanoma skin cancers (NMSCs), including basal cell carcinoma (BCC) and squamous cell carcinoma (SCC) [15, 38]. Unlike intermittent lifestyle exposures, UVB exposure is both unavoidable and cumulative across the lifespan, with dose intensity shaped by latitude, altitude, season, and environmental reflectivity. Although these ecological determinants are non-modifiable, behavioral and occupational factors including outdoor work, recreational sun exposure, photoprotection practices, and access to protective equipment substantially influence individual-level UVB dose and therefore represent important prevention targets [39, 40]. A joint World Health Organization and the International Labour Organization (WHO-ILO) global assessment estimated that in 2019, ~1.6 billion workers were exposed to solar UV radiation, contributing to 65,440 NMSC deaths worldwide. Notably, one-third of these deaths (around 18,960) and nearly half a million disability-adjusted life years were directly attributable to occupational UV exposure, an 88% increase since 2000 [41]. A key mechanistic link between UVB exposure and rising NMSC incidence is its ability to induce direct genotoxic and immunomodulatory effects. UVB generates DNA adducts and related photolesions that, when imperfectly repaired, accumulate as mutational signatures and transform into cancer [38, 42]. In parallel, UVB suppresses cutaneous immune surveillance by impairing antigen presentation and promoting local immunoregulatory signaling, thereby reducing the skin’s ability to eliminate transformed cells. This rising burden of UVB-driven NMSC, now one of the most frequently diagnosed cancers worldwide highlights persistent gaps in photoprotection practices and underscores preventable inequities shaped by occupational and socioeconomic factors. Modifiable behavioral and occupational determinants play a critical role in shaping individual UVB exposure and therefore represent important targets for prevention. Consistent sunscreen use, appropriate protective clothing, seeking shade during peak UV hours, and adherence to photoprotection guidelines can substantially reduce UV-induced skin damage. Occupational practices also strongly influence risk: outdoor workers in agriculture, construction, transportation, and informal labor frequently experience prolonged, unregulated UVB exposure, often without access to shade structures, protective gear, or routine skin surveillance. These modifiable factors underscore the need for stronger workplace safety policies, educational interventions, and equitable access to photoprotective resources to reduce preventable UV-related skin cancer risk [43–45]. These vulnerabilities may intersect with other risk modifiers such as age, immunosuppression, and metabolic disease, amplifying cumulative carcinogenic risk across decades of exposure. These early molecular and immunologic consequences of chronic UVB exposure help contextualize the epidemiological trends underlying the global rise in skin cancers, but further research is needed to fully elucidate the mechanistic links.

### Obesity, UV exposure, and shifting cancer risk dynamics

Regions with high obesity prevalence often coincide with areas of elevated UV exposure, for example, the U.S. South and Midwest, which also experience high summer UV indices. In 2024, all U.S. states and territories had an obesity prevalence of 25% or higher (at least 1 in 4 adults). Overall, the Midwest (35.9%) and South (34.5%) had the highest prevalence of obesity, followed by the West (30.2%) and the Northeast (30.3%). The coincidence of high obesity and high UV in some US regions does not necessarily imply that UV exposure causes obesity. These geographic correlations are associative and do not imply causality. Research indicates that the extreme temperatures, both very hot summers and very cold winters in these regions discourage physical activity, which is one of the major contributors to higher obesity rates [46]. Beyond geographic patterns, some studies suggest potential inverse associations between UV exposure and metabolic dysregulation (e.g., reduced weight gain or improved metabolic signaling), with proposed mechanisms such as UV-induced adipose browning. These findings are correlative in humans and require mechanistic and interventional confirmation to establish causality [47]. In another study, early-life exposures illustrate additional associative signals. Higher birth weight and substantial UV exposure in early childhood have been reported as independent observational correlates of melanoma diagnosed before age 30, supporting risk association and motivating sun-protective behaviors from infancy; however, real-world data on infant photoprotection practices are needed to clarify pathways [48]. Another study suggests that over the past 36 years in the US, the influence of cancer risk factors has shifted. Solar UVB exposure, once strongly linked to lower rates of many cancers, now shows a weaker inverse association with only about ten cancer types. Notably, cancers such as colorectal, renal, myeloma, and Non-Hodgkin Lymphoma no longer show clear protective patterns [49]. In contrast, diet-related factors tied to obesity and diabetes appear increasingly important. These population-level associations are hypothesis-generating and not causal; analytical designs beyond ecological comparisons, e.g., pooled cohorts, Mendelian randomization are needed to disentangle confounding and directionality. Also, more research is needed to understand how these factors interact and to guide public health strategies that can better reduce cancer risk [50]. While this ecological overlap does not establish causality, it underscores the potential for compound risk, shaped by individual factors such as skin phototype, occupation, mobility, and photoprotection behaviors. Understanding these interactions may help refine strategies for disease prevention and public health guidance.

### Interplay between UVB-induced DNA damage and obesity-related chronic inflammation

**Fig. 1:**
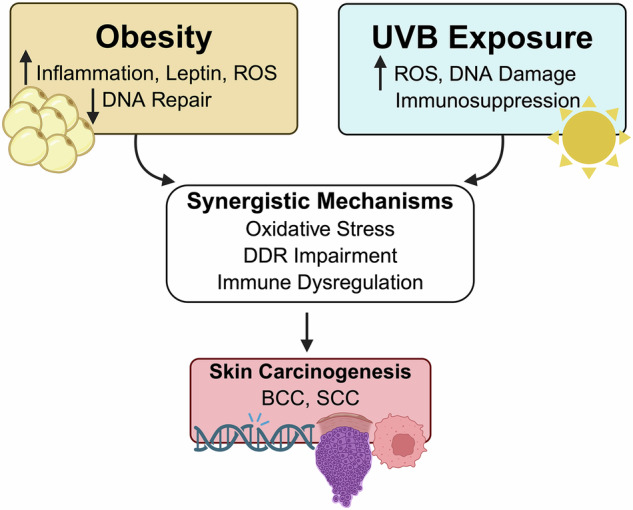
Synergistic mechanisms linking obesity and UVB exposure in skin carcinogenesis. Schematic representation of shared and synergistic pathways contributing to skin carcinogenesis. Obesity promotes chronic inflammation, elevated leptin, increased reactive oxygen species (ROS), and impaired DNA repair. UVB exposure induces ROS, DNA damage, and immunosuppression. These factors converge to amplify oxidative stress, disrupt DNA damage response (DDR), and cause immune dysregulation, collectively driving the development of basal cell carcinoma (BCC) and squamous cell carcinoma (SCC).

Obesity is no longer viewed as a passive accumulation of fat but as a dynamic, metabolically active condition that reshapes whole-body physiology through chronic low-grade inflammation [31, 51]. White adipose tissue functions as both an endocrine and immune organ, secreting adipokines, cytokines, and chemokines that drive metabolic and inflammatory remodeling [52]. As adipose depots expand, adipocyte hypertrophy and local hypoxia induce cellular stress and promote adipocyte death, leading to macrophage infiltration and the formation of a pro-inflammatory microenvironment. This milieu is characterized by elevated levels of TNF-α, IL-6, and MCP-1, activation of NF-κB and JAK/STAT signaling pathways, and consequent impairment of insulin sensitivity [53]. Concomitantly, obesity alters the adipokine profile, increasing pro-inflammatory leptin while reducing the insulin-sensitizing adipokine adiponectin. These changes are accompanied by a shift in immune cell composition toward pro-inflammatory M1 macrophages and Th1/Th17 effector T cells, together with a relative depletion of regulatory T cells and anti-inflammatory M2 macrophages [5, 54]. This chronic inflammatory state extends beyond metabolic and cardiovascular dysfunction to impair genomic maintenance and immune surveillance. Obesity-associated reductions in nucleotide excision repair (NER) capacity, together with features of T-cell exhaustion [55], may synergize with UVB-induced DNA damage and immunosuppression, thereby increasing vulnerability to skin carcinogenesis and potentially contributing to broader cancer risk (Fig. 1). In leptin-deficient *ob/ob* mice, UVB exposure induces markedly elevated levels of COX-2, prostaglandin E₂, and pro-inflammatory cytokines (TNF-α, IL-1β, IL-6), along with increased expression of proliferation and survival markers such as PCNA, PI3K, and phosphorylated Akt, compared with lean wild-type controls. These mice also exhibit heightened NF-κB/p65 activation and reduced UVB-induced apoptosis, indicating an exaggerated inflammatory and pro-survival response that may enhance susceptibility to UVB-driven inflammatory skin pathology and carcinogenesis [56]. Complementary studies demonstrate that obesity amplifies UVB-induced oxidative stress and photo-oxidative damage to lipids and proteins, accompanied by heightened MAPK and NF-κB activation. These effects appear to be mediated, at least in part, by reduced MAP kinase phosphatase activity in the skin of mice with obesity, providing early evidence that obesity confers greater susceptibility to UVB-driven oxidative injury [57]. Consistent with these findings, both obesity and aging have been linked to disruption of DNA repair pathways and increased metabolic and cancer risk; notably, individuals with obesity exhibit reduced double-strand break repair efficiency, with parallel support from preclinical models [58, 59]. Together, these findings suggest that obesity-associated oxidative signaling may potentiate UVB-induced tissue damage in experimental systems, while human data point to impaired repair capacity that could heighten susceptibility to UVB-induced genomic injury and carcinogenesis. However, prospective clinical studies directly connecting these repair deficits to incident UVB-driven skin cancers remain sparse and are needed to establish causality. Additional in vivo mechanistic work is likewise required to define how obesity shapes vulnerability to UVB-related photocarcinogenesis and photoaging compared with lean states and across different tumor types. UVB radiation itself exerts potent genotoxic and immunomodulatory effects that independently contribute to skin carcinogenesis [17, 60]. UVB photons induce cyclobutane pyrimidine dimers and 6-4 photoproducts while generating reactive oxygen species (ROS) that intensify oxidative stress and lipid peroxidation [61]. Beyond DNA damage, UVB impairs cutaneous immunity by depleting Langerhans cells, expanding regulatory T cells, and upregulating immunosuppressive mediators such as IL-10 and PGE₂, creating a tumor-permissive microenvironment [62–66]. Obesity establishes a pro-oxidative baseline through chronic inflammation, mitochondrial stress, and NADPH oxidase activity, raising resting ROS levels. Immune defenses are likewise compromised with obesity promoting T-cell exhaustion, dampens NK cell activity, and alters dendritic cell function [67]. Superimposed UVB-induced ROS can overwhelm antioxidant defenses (e.g., superoxide dismutase, glutathione peroxidase), leading to extensive oxidative injury and lipid peroxidation that fuel mutagenesis and inflammatory cascades [68]. At the genomic level, obesity has been linked to downregulation or functional impairment of NER components (e.g., XPA, XPC) and perturbations in p53 signaling, with emerging observational human evidence for reduced repair capacity and diminished double-strand break repair efficiency [64, 69]. Together, obesity and UVB create a hostile biological context that accelerates carcinogenesis via intersecting oxidative, genomic, and immune pathways. While these findings collectively support impaired genomic stability primarily on mechanistic and preclinical grounds, definitive clinical evidence tying obesity-associated DNA repair deficits to incident UVB-driven skin cancers remains limited and warrants prospective, biomarker-integrated human studies.

### Clinical and experimental evidence

Evidence from both animal models and human studies underscores the compound risk posed by obesity and UVB exposure. Murine models of diet-induced obesity (DIO) consistently demonstrate an increased incidence and multiplicity of UVB-induced skin tumors, accompanied by delayed resolution of UVB-induced erythema and epidermal hyperplasia [70, 71]. These models also reveal prolonged expression of DNA damage markers such as γH2AX following UVB exposure, suggesting impaired repair kinetics in the context of metabolic dysregulation [7]. In our unpublished studies, Nile Red staining of primary human dermal fibroblasts (HDFs) 24 h after UVB irradiation revealed increased intracellular lipid accumulation. Relative to untreated controls, UVB-exposed HDFs (30 mJ/cm²) displayed enhanced lipid synthesis, an effect attenuated by Everolimus (200 nM) but markedly amplified by Chloroquine (50 μM). These results suggest that promoting autophagy prior to UVB exposure limits lipid buildup, likely by enhancing cellular stress tolerance, whereas autophagy inhibition exacerbates UVB-induced lipid accumulation. Collectively, these findings raise the possibility that UVB-triggered lipid biosynthesis represents a protective metabolic adaptation or constitutes a mechanistic link between UVB exposure and metabolic alterations associated with obesity. Human observations parallel these findings: individuals with obesity exhibit higher rates of actinic keratoses and non-melanoma skin neoplasms in chronically sun-exposed areas, implicating obesity as a cofactor in photocarcinogenesis [72]. Reduced mobility in patients with obesity may further contribute to prolonged sun exposure of localized skin regions, while variations in UVB-induced erythema thresholds across body mass index (BMI) categories point to potential alterations in skin barrier properties and immune responsiveness [73]. Collectively, these experimental and clinical data support a biologically plausible link between obesity and heightened UVB sensitivity, reinforcing the need for integrated strategies that address both metabolic and environmental determinants of skin cancer risk.

### Emerging insights: UVB as a modulator of obesity

Paradoxically, recent research suggests that ultraviolet-B (UVB) radiation, traditionally viewed as a carcinogenic stressor, may exert beneficial systemic effects on metabolic health under controlled conditions [74]. Table 1 showing the comparative effects of obesity vs UVB on cutaneous & systemic pathways. UVB exposure stimulates cutaneous synthesis of vitamin D3, a secosteroid hormone with well-documented anti-inflammatory and insulin-sensitizing properties, which can improve glucose homeostasis and reduce chronic inflammation associated with obesity [75]. Beyond vitamin D pathways, experimental studies in murine models reveal that sub erythemal UVB exposure attenuates weight gain, decreases adipose tissue inflammation, and enhances insulin sensitivity, potentially mediated by nitric oxide (NO) release and other photoproducts that influence vascular tone and metabolic signaling [76]. Translational interest in these findings has led to clinical trials investigating narrowband UVB phototherapy as an adjunctive intervention for improving metabolic profiles in individuals with obesity and type 2 diabetes [77]. While these observations open intriguing therapeutic possibilities, they must be balanced against the well-established carcinogenic risks of UVB exposure, emphasizing the need for dose optimization, risk stratification, and long-term safety evaluation before clinical implementation. This duality-UVB as both a genotoxic agent and a potential metabolic modulator underscores the complexity of its biological effects and the importance of precision approaches in leveraging phototherapy for metabolic health.

**Table 1:** Comparative impacts of obesity and UVB exposure on cutaneous and systemic biological pathways.

Pathway	Obesity (evidence)	UVB (evidence)	Intersection	Key references
Oxidative stress	↑ ROS via mitochondrial dysfunction, NADPH oxidase, ER stress	↑ ROS; cyclobutane pyrimidine dimers (CPDs); secondary lipid peroxidation	Amplified oxidative injury; pro-tumor inflammation	[4, 78]
DNA repair/NER	Obesity is linked to DNA damage and reduced repair capacity (indirect)	NER (XPC/XPA/RPA) essential for excising UV lesions; CPD recognition limits	Potential NER vulnerability under metabolic stress	[55, 79]
Immune modulation	Leptin-driven Th1/Th17; T/NK dysfunction; impaired immunosurveillance	Langerhans cells → IL-10/Treg; local tolerance; dampened antigen presentation	Reduced detection/clearance of UV-mutated clones	[80–83]
Metabolic signals	Insulin resistance; NAFLD; systemic inflammation	NO & vitamin D mediate systemic effects; phototherapy modulates proteome	Cautious exploration of Phototherapy for metabolic modulation	[34, 84, 85]

This table delineates how obesity and UVB irradiation independently influence oxidative stress, nucleotide excision repair (NER) and broader DNA repair capacity, immune modulation, and metabolic signaling. It also highlights mechanistic intersections where these conditions may converge, synergize, or exacerbate one another’s effects, providing a framework for understanding shared vulnerabilities and compound pathophysiological outcomes.

### Public health implications and future directions

The co-occurrence of obesity and high ultraviolet B (UVB) exposure paradoxically constitutes a compound risk factor for skin cancer and other inflammation-driven disorders, highlighting the need for integrated, multifaceted public health strategies. Dermatologic screening should be prioritized for individuals with obesity, particularly those living in high-UV-index regions or engaged in outdoor occupations, as impaired DNA repair and immune surveillance may heighten vulnerability to UV-induced carcinogenesis. Public education campaigns must go beyond generic sun safety messages, tailoring interventions to populations with metabolic syndrome or limited mobility, who may experience prolonged localized sun exposure and reduced access to photoprotection resources. At the policy level, occupational health frameworks should incorporate UV protection measures such as shade structures, protective clothing, and sunscreen availability while simultaneously addressing obesity prevention through nutrition and physical activity programs. From a research perspective, future studies should explore mechanistic intersections between leptin signaling, vitamin D metabolism, nitric oxide pathways, and DNA damage response (DDR) efficiency to identify novel therapeutic targets. Additionally, clinical trials evaluating controlled phototherapy for metabolic modulation must balance potential benefits against long-term carcinogenic risks, emphasizing precision dosing and risk stratification. Ultimately, addressing this dual burden requires an interdisciplinary approach that integrates dermatology, endocrinology, occupational health, and behavioral science to mitigate the synergistic impact of obesity and UVB exposure on global health.

## Conclusion

Obesity and ultraviolet-B (UVB) radiation represent a unique convergence of metabolic and environmental stressors that collectively amplify the risk of inflammatory and neoplastic diseases. While traditionally considered independent risk domains, emerging evidence demonstrates that these factors intersect through shared biological pathways, including oxidative stress amplification, impaired DNA damage response, and immune dysregulation, creating a permissive environment for carcinogenesis. Experimental and clinical data support this synergistic relationship, highlighting obesity-driven inflammation and hormonal imbalance as modifiers of UVB-induced genotoxicity and immunosuppression. At the same time, paradoxical findings on UVB-mediated metabolic modulation underscore the complexity of its systemic effects and the need for precision approaches in leveraging phototherapy. Addressing this dual burden requires integrated strategies that combine dermatologic screening, tailored sun safety education, metabolic risk management, and occupational health protections. Future research should prioritize mechanistic studies on DNA repair efficiency, immune surveillance, and phototherapy safety, alongside clinical trials that balance potential metabolic benefits against carcinogenic risks. Ultimately, a deeper understanding of the obesity-UVB interaction offers an opportunity to refine prevention, improve risk stratification, and develop innovative interventions in the face of two converging global health challenges obesity and skin cancer.
